# Responding to the evidence for the management of severe malaria

**DOI:** 10.1111/j.1365-3156.2011.02810.x

**Published:** 2011-06-20

**Authors:** Nathan P Ford, Martin de Smet, Kavitha Kolappa, Nicholas J White

**Affiliations:** 1 Médecins Sans FrontièresBrussels, Belgium; 2 Centre for Infectious Disease Epidemiology and ResearchCape Town, South Africa; 3 Johns Hopkins University School of MedicineBaltimore, MD, USA; 4 Mahidol Oxford Tropical Medicine Research Unit, Mahidol UniversityBangkok, Thailand

**Keywords:** severe malaria, quinine, artesunate, treatment

Humanity owes a great debt to quinine. Cinchona alkaloids have been used to treat malaria for hundreds of years after the arrival of cinchona bark in Europe in the 17th century, where it was mixed with rose leaves, lemon juice and wine to treat the malarious patients of Essex (Butler *et al.* 2010). While quinine has been largely replaced by more effective and better-tolerated drugs in the treatment of uncomplicated malaria, it remains the standard treatment for severe malaria in many countries. In Africa, where over 90% of the estimated 781 000 malaria-related deaths in 2009 occurred, quinine is first-line therapy for severe malaria in almost all countries (WHO 2010).

Recent evidence shows that it is time to replace quinine for severe malaria as well. A large randomized trial conducted in Asia in 2005 found that parenteral artesunate reduced overall mortality by 39% compared with quinine (Dondorp *et al.* 2005). As a result, in 2006 WHO recommended artesunate as the treatment of choice for adults, but considered there was insufficient evidence to extend this recommendation to children in Africa. That evidence came in late 2010, from the largest ever study of severe malaria (5425 children across Africa), which found that artesunate reduced mortality by 22.5% compared with quinine (Dondorp *et al.* 2010). Importantly, there was no evidence in this or previous studies of an increase in neurological sequelae in survivors. These results were confirmed by a recent Cochrane meta-analysis that found an overall mortality reduction of 39% among adults and 24% among children compared to quinine (Sinclair *et al.* 2011), an identical finding to the meta-analysis accompanying the African trial (Dondorp *et al.* 2010).

So what now? WHO has just changed its guidelines to put artesunate as the treatment of choice for severe malaria everywhere (WHO 2011), and these recommendations need to be disseminated to all relevant malaria actors to support national guideline change where needed. To date, only one African country (Nigeria) has revised its guidelines to include artesunate for severe malaria, and only as an alternative treatment (WHO 2010). Given the long history of quinine use, the dissemination of national guidelines will need to be accompanied by training to help shift health provider habits and personal convictions. All this needs to be properly supported, both by international donors and through technical advice from WHO’s regional offices.

The challenge in translating evidence into practice should never be underestimated, particularly when it comes to malaria treatment, as recent history shows. In 2000, WHO recommended a policy shift in the management of uncomplicated malaria from chloroquine to artemisinin-based combination therapies (ACTs) because of high levels of chloroquine resistance. Yet, despite substantial evidence supporting the superior efficacy of ACTs, international donors and ministries of health in malaria-endemic countries continued to support chloroquine use for several years, mainly because chloroquine was a much cheaper drug (Attaran *et al.* 2004).

Similarly, the higher unit price of artesunate is likely to be a barrier. Cost effectiveness studies, however, show that when mortality and associated costs such as reduced side-effect management and hospitalization are considered, artesunate is cost effective (Lubell *et al.* 2009, 2011). Furthermore, the cost of artesunate may fall as demand increases. Nevertheless, for policy and practice to change in the short-term, additional international funding support is needed.

Another important potential concern is the limited availability of quality-assured sources of artesunate. Currently, only one source of injectable artesunate has been pre-qualified by WHO, and malaria control programmes may be reluctant to make the switch as long as consistency in supply is uncertain. In addition, quinine production represents an important economic activity in a number of malaria-endemic countries; in Burundi, for example, quinine is one of the few drugs manufactured in-country, making it more popular and more easily accessible than imported antimalarial drugs (Amuasi *et al.* 2011). However, artemisinin, the raw material for artesunate, is increasingly being produced in Africa, including in Kenya, Tanzania and Uganda (http://www.artepal.org), and as demand grows, supply should logically follow.

Finally, it can be anticipated that calls for local evidence may be made by national governments out of concern that studies performed in other contexts may not apply to their setting. MSF faced similar challenges when trying to move from chloroquine to ACT (Guthmann *et al.* 2008). While this may initially appear reasonable, the evidence to date is broadly generalizable and the latest Cochrane review concludes that further research to assess the efficacy of artesunate versus quinine is unnecessary (Sinclair *et al.* 2011). Patients should only be subjected to experimental trials if there is real uncertainty about which drug is better (CIOMS 2002), and while operational research may help guide implementation, it would clearly be unethical to delay implementation and subject patients to further drug effectiveness studies.

Global funding for malaria control is already insufficient (Snow *et al.* 2010), and in the current economic climate, donors may be reluctant to support a switch to a more expensive treatment. However, replacing quinine with artesunate is a clear cut intervention that has the potential to save nearly 200 000 lives each year and the total annual cost of providing artesunate for treating all cases of severe malaria worldwide would likely be less than $US 50 million (MSF 2011). For African countries to make the switch, strong international support will be required to provide additional funds to support drug procurement and training costs and send a clear message to manufacturers that quality sources of artesunate are needed.
